# Research Progress on Regulation of Immune Response by Tanshinones and Salvianolic Acids of Danshen (*Salvia miltiorrhiza* Bunge)

**DOI:** 10.3390/molecules29061201

**Published:** 2024-03-07

**Authors:** Jiawen Tang, Xueying Zhao

**Affiliations:** School Basic Medical Sciences, Heilongjiang University of Chinese Medicine, 24 Heping Road, Harbin 150040, China; tjw18045448324@126.com

**Keywords:** Danshen, *Salvia miltiorrhiza* Bunge, tanshinones, salvianolic acids, immune response

## Abstract

As one of the traditional Chinese herbs, Danshen (*Salvia miltiorrhiza* Bunge) has been widely studied and widely used in the treatment of cardiovascular, cerebrovascular, and other immune diseases. Tanshinones and salvianolic acids isolated from Danshen are considered to be the main components of its biological activity and pharmacology that play important roles in increasing the index of immune organs, regulating the number and function of immune cells, and releasing immunoreactive substances. Especially tanshinone IIA, cryptotanshinone, salvianolic acid B, and rosmarinic acid show good biological activity in treating rheumatoid arthritis, some immune-mediated inflammatory diseases, psoriasis, and inflammatory bowel disease. In order to understand their pharmacological effects and provide references for future research and clinical treatment, the regulation of immune response by tanshinones and salvianolic acids is summarized in detail in this paper. In addition, the challenges in their pharmacological development and the opportunities to exploit their clinical potential have been documented.

## 1. Introduction

With the rapid pace of modern life, more and more people are in a state of sub-health or even pathology. Normal immune response plays an important role in protecting the body, such as tumor prevention and control and anti-inflammatory response. Acquired immune response is essential for immunity to inflammation, infection, and tumor [1]. Both innate and acquired immunity can regulate the immune response of the body. Many traditional Chinese medicines have immune-enhancing effects [2].

Danshen as a traditional Chinese medicine was first published in “Shennong’s Herbal Classic of the Materia Medica” and is the dried root and rhizome of *Salvia miltiorrhizae* Bunge of the Labiatae family, and its surface is reddish-brown or dark reddish-brown, rough with longitudinal wrinkles. Danshen is found in many countries of the world and has been used for thousands of years to treat many diseases. Danshen is bitter in taste and slightly cold in nature and belongs to the heart and liver meridians. According to traditional Chinese medicine, Danshen is effective in clearing the heart and removing vexation, activating blood circulation, eliminating blood stasis, cooling the blood, eliminating carbuncles, and clearing menstruation and relieving pain [3]. Moreover, Danshen is often made into various dosage forms for the treatment of cardiovascular and cerebrovascular diseases [4]. Up to now, more than 50 compounds from aqueous extracts and over 80 lipophilic compounds have been isolated from Danshen [5]. In addition, a large number of pharmacological studies showed that Danshen and its main components such as fat-soluble tanshinones (including tanshinone IIA, tanshinone I, cryptotanshinone, and dihydrotanshinone I, etc.) and water-soluble salvianolic acids (including salvianolic acid A, salvianolic acid B, and rosmarinic acid, etc.) also exhibit other pharmacological activities such as anti-inflammatory, antioxidant, anti-tumor, and anti-fibrosis activity and modulation of the immune response [5]. 

For instance, salvianolic acids have been found to be useful in ameliorating Alzheimer’s disease (AD) [6,7]. Chong et al. briefly summarized the studies regarding the effects of Danshen components on the major characteristics of AD and explored their possibility for the treatment of AD [8]. Furthermore, salvianolic acid A, tanshinone I, tanshinone IIA, and cryptotanshinone showed protective activities on amyloid precursor protein processing, tau hyperphosphorylation, mitochondria dysfunction, as well as abnormal autophagy against AD. Among them, salvianolic acid B showed more potential applications because it appears to reduce four characteristics of AD [8]. Marrelli et al. investigated the inhibitory effects of tanshinone IIA and cryptotanshinone on pancreatic lipase [9]. Liu et al. found that tanshinones and salvianolic acids can improve non-alcoholic fatty liver through different pathways and mechanisms [10]. Wang showed that a Danshen tablet and its eight active components (dihydrotanshinone I, tanshinone I, tanshinone IIA, cryptotanshinone, danshensu, salvianolic acid A, salvianolic acid B, and salvianolic acid C) potently inhibited the metabolism of rivaroxaban in rat and human liver microsomes [11]. Wu et al. demonstrated that tanshinones and phenolic acids synergistically treat coronary heart disease through the same targets of the same biological process and different targets of the same biological process [12]. Yan et al. found that tanshinone IIA may be the main active component of compound Danshen dropping pills (CDDPs) in the treatment of high-altitude heart disease [13]. Tanshinol, salvianolic acid B, and tanshinone IIA were the pivotal anti-inflammatory ingredients in CDDPs, and they ameliorated post-ischemic myocarditis [14]. Qu et al. showed that nine compounds, including danshensu, lithospermic acid, rosmarinic acid, salvianolic acid B, salvianolic acid A, and salvianolic acid C, can also improve myocardial ischemic injury [15]. Wan et al. demonstrated that the combination of Danshen and Honghua exhibited a protective effect on rats after cerebral ischemia–reperfusion injury [16]. Chen et al. demonstrated that caffeic acid, danshensu, lithospermic acid, rosmarinic acid, salvianolic acid A, salvianolic acid B, salvianolic acid C, salvianolic acid D, and 3,4-Dihydroxybenzaldehyde in a Danshen injection can induce autophagy in podocytes to alleviate nephrotic syndrome [17]. Zhang et al. screened 11 components of the Danshen–Honghua herbal pair; among them, salvianolic acid B was one of the compounds with the highest content and proved their activity against vascular dementia [18]. Qin et al. showed that the effective components (salviolone and tanshinone IIA) of the Danshen–Guizhi drug pair in the treatment of ovarian cancer regulated proliferation, apoptosis, and tumor immunity [19]. Sustained-release preparations can markedly prolong the in vivo coexistence of tanshinone IIA, tanshinol, protocatechuic aldehyde, and salvianolic acid B in Danshen compared to the commercial Danshen capsules to enhance their overall effects [20]. Tanshinone IIA isolated from Danshen can alleviate cisplatin-induced acute kidney injury through the regulation of PXR/NF-κB signaling [21]. 

In addition, some researchers reviewed the biological activity and pharmacology of the isolated active constituents of Danshen [22,23,24,25,26,27]. For example, Li et al. summarized the pharmacological potential of cryptotanshinone and comprehensively described the molecular pathway mechanisms implicated in the effects of cryptotanshinone on the treatment of different diseases [22]. Recent advances and future directions in the anti-tumor activity of cryptotanshinone A were reported by Ashrafizadeh et al. [23]. Lin et al. found that dihydroisotanshinone I can inhibit the growth, ferroptosis, and apoptosis of breast carcinoma cells and inhibit the final tumor volume without adverse effects [24]. Guo et al. reviewed the role of tanshinone IIA isolated from Danshen in different diseases and its mechanism [25]. A meta-analysis showed the effectiveness of tanshinone IIA in brain protection in animal models of cerebral ischemic injury [26]. Du et al. presented a review of the chemical and pharmacological research on the polyphenol acids isolated from Danshen [27]. 

In general, most studies on Danshen focus on the treatment of cardiovascular and cerebrovascular diseases. However, there is no systematic review on the pharmacological effects of Danshen and its main constituents on regulating the immune response and immune-related diseases. In this review, we mainly summarize the pharmacological effects of tanshinones and salvianolic acids in improving the immunomodulatory effect and the treatment of autoimmune diseases and common immune-mediated inflammatory diseases. Moreover, the pathways and mechanisms of different compounds of tanshinones and salvianolic acids in regulating the immune response were analyzed and compared in detail. Furthermore, we expect to provide a theoretical basis for the further development and utilization of Danshen in clinical practice. 

## 2. Main Components of Danshen 

A variety of chemical constituents such as tanshinones, salvianolic acids, polysaccharides, flavonoids, and volatile oils, etc., have been isolated and identified from Danshen [28] using traditional extraction methods (decoction method, infusion process maceration, continuous reflux extraction, etc.) and new technologies (semi-bionic extraction method, ultrasonic extraction, and supercritical fluid extraction, etc.). Among these constituents, tanshinones and salvianolic acids are considered as the main components having pharmacological activity to regulate the immune response.

### 2.1. Tanshinones

**Figure 1:**
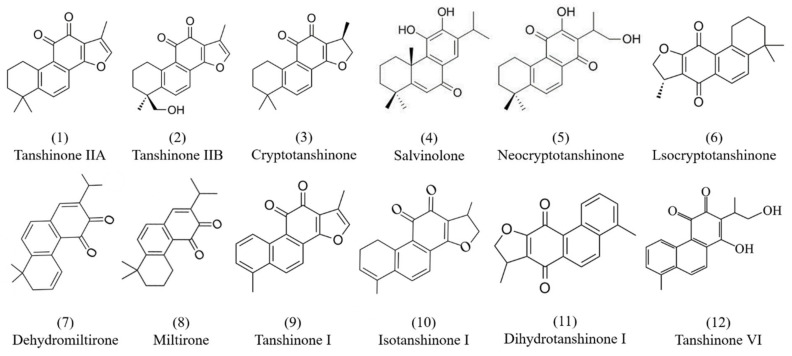
Chemical structures of tanshinone components isolated from Danshen.

Tanshinones are mostly diterpenoids in nature, and all of them contain a p-quinone or o-quinone structure, are fat-soluble, and are mainly synthesized and accumulated in the peripheral cortex of the roots of Danshen [29]. In fact, no less than 30 diterpenoid tanshinone components have been isolated and identified from Danshen, such as tanshinone IIA (**1**), tanshinone IIB (**2**), cryptotanshinone (**3**), salvinolone (**4**), neocryptotanshinone (**5**), lsocryptotanshinone (**6**), dehydromiltirone (**7**), miltrione (**8**), tanshinone I (**9**), isotanshinone I (**10**), dihydrotanshinone I (**11**), and tanshinone VI (**12**), etc. [30,31,32]. The chemical structures of the major tanshinone components are shown in Figure 1. In addition, Roth et al. studied the purity and stability of tanshinone I (**9**), tanshinone IIA (**1**), cryptotanshinone (**3**), and dihydrotanshinone I (**11**) using liquid chromatography–diode array detection (LC-DAD) and liquid chromatography–mass spectrometry (LC-MS) analyses. Tanshinone I (**9**) and tanshinone IIA (**1**) were the most relevant bioactive tanshinones in Danshen, with no cytotoxic and genotoxic effects [33]. Currently, the extraction method for tanshinones from Danshen is inefficient, and Wei et al. showed that combining transgenic technology with elicitor Ag+, yeast extract (YE), and methyl jasmonate (MJ) treatment can effectively increase the level of tanshinones [34]. Most of the tanshinones have been used to study pharmacological activity, such as cryptotanshinone (**3**) [22,23], tanshinone IIA (**1**) [25], etc.

### 2.2. Salvianolic Acid 

**Figure 2:**
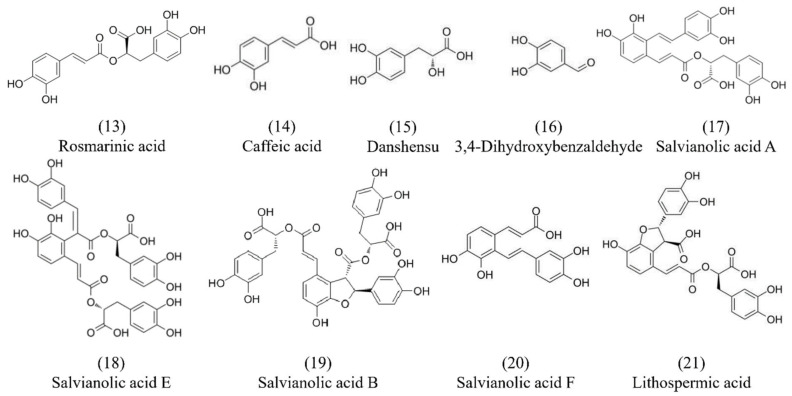
Chemical structures of salvianolic acid components from Danshen.

Salvianolic acid is also one of the main chemical components isolated from Danshen which is water-soluble and mainly synthesized and accumulated in the phloem and xylem of its root. To date, more than 30 species of salvianolic acid have been isolated from Danshen, such as rosmarinic acid (**13**), caffeic acid (**14**), danshensu (**15**), 3,4-dihydroxybenzaldehyde (**16**), salvianolic acid A (**17**), salvianolic acid E (**18**), salvianolic acid B (**19**), salvianolic acid F (**20**), and lithospermic acid (**21**), etc. [35,36]. The chemical structures of the major constituents of salvianolic acids are shown in Figure 2. Most of the salvianolic acids can be regarded as derivatives of caffeic acid (**14**) [37]. Additionally, salvianolic acids are the main water-soluble compounds, among which salvianolic acid A (**17**) and salvianolic acid B (**19**) are the most abundant components in Danshen. Ho et al. summarized the cardiovascular protective effect of salvianolic acids and the multiple mechanisms of these small compounds in terms of reactive oxygen species (ROS) scavenging ability, leukocyte–endothelial adhesion regulation, inflammation inhibition, and immune modulation [38]. Polyphenol acids isolated from Danshen showed special bioavailability, pharmacodynamics, and pharmacokinetics. Du et al. summarized the chemical and pharmacological research on the polyphenol acids isolated from Danshen [27]. Fan et al. showed that the components of salvianolic acids in full dried slices were higher than in traditional slices based on liquid chromatography–high-resolution mass spectrometry technology, which accumulated more effective ingredients [39]. 

Because tanshinones and salvianolic acids were the main ingredients isolated from Danshen, the researchers studied their biological activity and pharmacology in many diseases. So, we are going to focus on the progress of tanshinones and salvianolic acids in the immune response, providing references for the development of new drugs from Danshen to regulate the immune system and improve immune system diseases in the future.

## 3. Effects of Tanshinones on the Immune System

Several studies have shown that different doses of Danshen powder have different immunomodulatory effects. Thirty-five different compounds were identified in Danshen powder using HPLC and LC-MS/MS analysis, including tanshinone IIA (**1**), cryptotanshinone (**3**), tanshinone I (**9**), salvianolic acid A (**17**), and salvianolic acid B (**19**), etc. Danshen at a concentration of 0.5% reduced serum immunoglobulin E (IgE) production in BALB/c mice; 1% Danshen promoted cellular immunity; both 0.5% and 1% Danshen inhibited hepatic and splenic oxygen radical production and hepatic nitric oxide (NO) production; moreover, 2% Danshen improved the host resistance to Listeria monocytogenes (LM), increased peripheral blood mononuclear cells and natural killer (NK) cells, and decreased the production of interleukin-1β (IL-1β) and NO [40]. After reviewing a large amount of the literature, it was found that tanshinone IIA (**1**), cryptotanshinone (**3**), tanshinone I (**9**), and dihydrotanshinone I (**11**), etc., can affect the immune system, such as by increasing the index of immune organs, regulating the number and function of immune cells, and modulating the release of immunoreactive substances (Table 1).

### 3.1. Increasing Immune Organ Index

The index of immune organs is one of the most important indicators of the body’s immune function. Tanshinones do not directly regulate the bone marrow, but they play an important role in the regulation of a variety of immune cell subpopulations contained in the bone marrow that have important and unique functions and in immune cell homeostasis.

Di et al. in studying the anti-tumor effects of total tanshinone (TT) and nanoparticles of tanshinone (NT) on U14 cervical cancer mice found that both of them not only increased the tumor suppression rate but also increased the thymus and spleen indices and improved the immune function of the mice, and the effect of NT was better than that of TT [41]. When Zhang et al. investigated the inhibitory effect of tanshinones on the growth of hepatocellular carcinoma cells in vitro and in vivo, they found that as the dose of tanshinones increased, the thymus and spleen index of mice also became higher and higher, indicating that tanshinones could improve the immune function and that the anti-tumor effect was probably related to the improvement in immunity [42]. Furthermore, tanshinone IIA (**1**) also had an anti-tumor effect on stressed mice, which increased the thymus index and improved the immune function by increasing the expression of interleukin-2 (IL-2) and IL-2R [43]. Cryptotanshinone (**3**) had a bi-directional immunomodulatory effect, which enhanced the immune function of thymocytes at a low concentration and negatively inhibited the immune function of hyperactive splenocytes at a high concentration, in a concentration- and function-dependent manner [44]. In addition, it was found that both 50 and 100 mg/kg of cryptotanshinone (**3**) could increase the thymic and splenic indices and ameliorate inflammation and joint destruction in arthritic rats in vivo by regulating the immune function [45]. The above studies demonstrate that tanshinones can not only increase the immune organ index but also play an anti-tumor and anti-inflammatory role by improving the immune function.

### 3.2. Regulating the Number and Function of Immune Cells

When an inflammatory response occurs, the local inflammation will be infiltrated by a large number of immune cells such as macrophages, neutrophils, and T cells, which are activated to release inflammatory factors such as TNF-α, IL-6, IL-1β, and IL-10, leading to the aggravation of the inflammatory response. The anti-inflammatory mechanism of Danshen includes the modification of humoral and non-specific immunity and the alleviation of hepatotoxicity [46]. The immune response to tumor is a complex process [47]; dendritic cells (DCs) and T cells are an indispensable part in the adaptive immune response to tumors [48]. Wong et al. reported that Yunzhi (50 mg/kg body weight)–Danshen (20 mg/kg body weight) capsules can regulate and enhance cellular immunity by increasing the number and function of T helper (Th) cells without causing inflammation [49]. In addition, Yunzhi–Danshen prescriptions containing Danshen had a positive effect on the immune function of nasopharyngeal carcinoma [50], breast cancer [51], and liver cancer [52] patients. 

#### 3.2.1. Effect on Neutrophils

Neutrophils, as sentinel cells in mucosal immunity, play a key role in host resistance to infection, but the inappropriate activation of neutrophils can lead to autoimmune and inflammatory diseases [53]. Tanshinone IIA (**1**) promoted the spontaneous apoptosis and reverse migration of neutrophils in a neuromyelitis optica (NMO) mice animal model, allowing neutrophils to be cleared from the site of injury and reducing inflammation [54]. Wang et al. found that cryptotanshinone (**3**) can significantly reduce the number and infiltration of total cells, neutrophils, and eosinophils in bronchoalveolar fluid, decrease airway resistance in mice, and attenuate inflammation to treat asthma through the inhibition of the signal transducer and activator of transcription 3 (STAT3) signaling pathway, which in turn can inhibit tumor necrosis factor-like weak inducer of apoptosis and transforming growth factor β1 (TGF-β1) signaling crosstalk [55]. The investigation by Zhao et al. demonstrated that dihydrotanshinone I (**11**) suppressed the formation of neutrophil extracellular traps (NETs) and ameliorated NET-induced metastasis, thereby inhibiting lung metastasis of breast cancer [56]. In addition, some scholars explored the anti-inflammatory properties of tanshinone I (**9**), isotanshinone I (**10**), and their analogous compounds using a zebrafish model, and the results showed that tanshinone I (**9**) and some compounds could reduce initial neutrophil recruitment, promote neutrophil inflammation subsidence, and increase neutrophil apoptosis [57].

#### 3.2.2. Effect on Macrophages and DCs

Macrophages are tissue-resident or infiltrating immune cells that are essential for innate immunity, normal tissue development, homeostasis, and the repair of damaged tissues [58]. Don et al. confirmed that cryptotanshinone (**3**) inhibited the migration of macrophages and the activation of phosphatidylinositol 3-kinase (PI3K), thereby reducing the phosphorylation of protein kinase B (AKT) and extracellular regulated protein kinases 1 and 2 (ERK1/2) for anti-inflammatory purposes [59]. Han et al. found that cryptotanshinone (**3**) inhibited the growth of mouse hepatocellular carcinoma (Hepa1-6) cells by inducing apoptosis through blocking the Janus kinase 2 (JAK2)/STAT3 signaling pathway and activated macrophages and polarized mouse bone marrow-derived macrophages towards a classically differentiated macrophage (M1) phenotype in vitro through the toll-like receptor (TLR) 7/myeloid differentiation factor 88 (MyD88)/nuclear factor-κ gene binding (NF-κB) signaling pathway [60]. Moreover, cryptotanshinone (**3**) also promoted the activation of tumor-infiltrating macrophages and dendritic cells (DCs), the induction of anti-tumor T-cell responses, and the infiltration of effector/memory CD8+ T cells in tumor tissues, which had the dual ability to inhibit multiple tumor growth and promote anti-tumor immune responses. In addition, cryptotanshinone (**3**) was able to promote the production of tumor necrosis factor (TNF)-α, IL-1β, and IL-12 by DCs without inducing the production of IL-10, preferentially promoting the immune response of type 1 helper (Th1) cells, and had the unique dual ability to inhibit the proliferation of lung cancer cells and induce the maturation of DCs [61]. Chen et al. certified that tanshinone IIA (**1**) isolated from Danshen could inhibit microRNA 375 (miR-375), activate Krüppel-like factor 4 (KLF4), and enhance macrophage autophagy and selectively differentiated macrophage (M2) polarization by mediating internal immune mechanisms [62]. Li et al. also found that tanshinone IIA (**1**) played a potential role in anti-atherosclerotic activity, which could inhibit DC-mediated adaptive immunity. Moreover, tanshinone IIA (**1**) was also able to inhibit the DCs’ maturation, decrease the expression of inflammatory factors IL-1 and IL-12, and weaken their ability to stimulate T-cell proliferation and cytokine secretion [63]. Dihydrotanshinone I (**11**) significantly reduced TNF-α, IL-6, and IL-1β released in LPS-stimulated mouse leukemia cells of monocyte macrophages (RAW264.7 cells, THP-1 cells) and myeloid-derived macrophages and inhibited NF-κB transcription and expression as well as the nuclear translocation of nuclear factor-κBp65 (NF-κBp65), acting as an anti-inflammatory agent [64]. 

#### 3.2.3. Effect on Lymphocytes

Lymphocytes are an important part of the body’s immune function and play an important role in the anti-infection, anti-tumor, and other immune processes. Generally, they are mainly categorized into T lymphocytes, B lymphocytes, and NK cells. Gao et al. found that tanshinone IIA (**1**) reversed sepsis-induced reductions in CD4+ and CD8+ T-cell populations, improved T-cell function in abdominal sepsis, balanced Th1/Th2 cells, and ameliorated immunosuppression in polymicrobial sepsis [65]. In addition, tanshinone IIA (**1**) inhibited the activation of NLR family pyrin domain-containing (NLRP) 3 inflammasome and the differentiation of Th17/regulatory (Treg) cells, attenuating myocardial injury and improving systemic and local inflammatory responses [66]. The results given by Zhang et al. showed that dihydrotanshinone I (**11**) reduced the number and infiltration of total cells, neutrophils, lymphocytes, and macrophages in the bronchoalveolar lavage fluid (BALF) of mice with cigarette smoke (CS)-induced pneumonia, improved the balance of Th1/Th2 cells, regulated the immune response of Th cells, inhibited the activation of inflammatory transcription factors STAT1 and STAT3, and reduced the levels of interferon (IFN)-γ and IL-17A and the expression of IL-4 and IL-13, thus alleviating CS-induced inflammation in the lung [67]. Some scholars examined the effects of tanshinone IIA (**1**) and cryptotanshinone (**3**) on the differentiation of NK cells in vitro, and the results indicated that both could promote the IL-15-induced differentiation of NK cells to enhance anti-tumor immune responses through activating the p38 mitogen-activated protein kinase (p38 MAPK) signaling pathway [68]. Kang et al. found that tanshinones had a potent inhibitory effect on the activation of IL-12 and IFN-γ production from Th1 cells, and dehydromiltirone (**7**) was more effective than tanshinone I (**9**) and tanshinone IIA (**1**) [69]. Thus, these results may explain the anti-inflammatory effect of tanshinones, confirming the speculation that tanshinones can be used in the treatment of immunological diseases dominated by Th-1-derived cytokine responses [69].

By summarizing the above research, it can be concluded that tanshinone IIA (**1**), cryptotanshinone (**3**), tanshinone I (**9**), and dihydrotanshinone I (**11**) have special effects on the number and function of immune cells, as well as the release of immune active substances, which can not only be used to alleviate the body’s inflammatory response but also have an anti-tumor immune effect, treating a variety of diseases.

**Table 1:** Effects of tanshinones on the immune system.

Components	Immune Organ	Immune Cells	Immune Active Substance	References
Tanshinone IIA (**1**)	Thymus			[43]
		Macrophages	↓IL-1, IL-12	[62]
		DCs		[63]
		T cells		[65]
		Th17/Treg		[66]
		NK cells	↑IL-15	[68]
		Th1 cells	↓IL-12, IFN-γ	[69]
Cryptotanshinone (**3**)	Thymus			[44]
	Spleen			[45]
	Thymus			
		Neutrophils		[55]
		Macrophages		[59,60]
		Macrophages	↑TNF-α, IL-1β, IL-12	[61]
		DCs	↓IL-10	
		NK cells	↑IL-15	[68]
Dihydrotanshinone I (**11**)		Neutrophils		[56]
		Macrophages	↓TNF-α, IL-6, IL-1β, ↓NF-κB	[64]
Tanshinone I (**9**)		Neutrophils		[57]
		Th1 cells	↓IL-12, IFN-γ	[69]
Dehydromiltirone (**7**)		Th1 cells	↓IL-12, IFN-γ	[69]

↑ means increase; ↓ means decrease.

### 3.3. Improving Autoimmune Diseases 

As displayed in Table 2, the main components of Danshen, tanshinone IIA (**1**), cryptotanshinone (**3**), and dihydrotanshinone I (**11**), have been found to play therapeutic roles in targeting the pathogenesis of the autoimmune diseases (AIDs).

#### 3.3.1. Effect on Rheumatoid Arthritis

The study confirmed that tanshinones can exert bone remodeling and bone protective effects by inhibiting osteoclastogenesis/bone resorption and promoting osteoblastogenesis/bone formation [70]. Tanshinones isolated from Danshen were able to ameliorate peroxidative damage, adjust the level of serum superoxide dismutase (SOD) and malonaldehyde (MDA), and inhibit the secretion of IL-6 and TNF-α, thus attenuating rheumatoid arthritis (RA) [71]. Cryptotanshinone (**3**) induced the apoptosis of fibroblast-like synovial cells in rheumatoid arthritis (RA-FLSs) through the reactive oxygen species-mediated Akt, mitogen-activated protein kinase (MAPK), and STAT3 signaling pathways. What is more, it could improve Th17/Treg cell balance through the inhibition of STAT3 acetylation and NF-κB activation via P300 proteins. In addition, cryptotanshinone (**3**) could downregulate the secretion of inflammatory factors and significantly reduce the production and activity of metalloproteinase (MP)-9 or reduce osteoclastogenesis by inactivating the NF-κB and extracellular regulated protein kinase (ERK) signaling pathways in bone marrow-derived macrophages to alleviate RA [72,73,74,75]. 

It was shown that tanshinone IIA (**1**) (1 and 5 μM) promoted osteoblast differentiation in both early and late stages of mouse bone marrow mesenchymal stem cells (BM-MSCs), whereas it inhibited their proliferation and differentiation at 20 μM [76]. Tanshinone IIA (**1**) also treated osteoclastic-mediated bone resorption by preventing osteoclast formation, and it is a potential drug candidate for the treatment of bone-related diseases [77]. Some studies found that tanshinone IIA (**1**) ameliorated RA mainly by promoting the apoptosis of RA-FLSs through the blockade of the cell cycle in the G2/M phase and the mitochondrial pathway to regulate the pro/anti-apoptotic protein expression [78]; upregulating the long non-coding RNA growth arrest-specific 5 (IncRNA GAS5) [79]; activating the phosphatidylinositol 3-kinase (PI3K)/AKT, MAPK, and AKT/mechanistic target of rapamycin (mTOR) and hypoxia-inducible factor-1 (HIF-1) signaling pathways [80]; and inhibiting the growth cycle transition of RA-FLSs, resulting in the blockage of the G2/M phase and the alteration of cell cycle progression [81]. Meanwhile, tanshinone IIA (**1**) reduced the viability of RA-FLSs in a dose- and time-dependent manner and blocked their migration and invasion in vitro [78]. Other studies showed that tanshinone IIA (**1**) could significantly inhibit the secretion of TNF-α, IL-6, and IL-17 in the peripheral blood of patients with LPS-induced RA by modulating the activity of neutrophils or β-arrestin 2, leading to the attenuation of inflammatory cell infiltration, ankle/knee swelling, synovial hyperplasia, and synovial tissue erosions, etc. [81,82]. Kim et al. demonstrated that tanshinone IIA (**1**) not only inhibited receptor activator of nuclear factor-κB (RANKL)-dependent osteoclast maturation by reducing cell fusion but also reduced the bone resorption activity of mature osteoclasts, inhibiting osteoclast differentiation by 79.70%, 93.62%, and 100% with 5, 10, and 20 μg/mL of tanshinone IIA (**1**), respectively [83]. 

In conclusion, it was concluded that the main role of tanshinone IIA (1) in improving RA was to inhibit the differentiation and maturation of RA-FLSs and osteoclasts through different pathways, as well as to reduce the release of inflammatory factors. In addition, the use of tanshinone IIA (**1**) iontophoresis for the treatment of RA was more long-lasting than other routes of administration [84]. Compared with cryptotanshinone (**3**), tanshinone IIA (**1**) can be found to alleviate RA through more pathways and more complex mechanisms. Tanshinone IIA (**1**) induced RA-FLS apoptosis by regulating the expression of procaspase 3/9 and caspase 3/9 protein [78]. Therefore, tanshinone IIA (**1**) may be more effective in treating RA. 

#### 3.3.2. Effect on Multiple Sclerosis

Many studies confirm that tanshinone IIA (**1**) is a major component of tanshinone and effective in alleviating multiple sclerosis (MS) [85,86,87]. The experimental results of Yan et al. demonstrated that tanshinone IIA (**1**) significantly reduced the number of CD4+ T cells, CD8+ T cells, microglia, and macrophages in mice and attenuated experimental autoimmune encephalomyelitis (EAE) by downregulating the IL-23 and IL-17 levels and reducing the infiltration of immune cell populations [85]. Yang et al. found that tanshinone IIA (**1**) attenuated motor dysfunction, ameliorated inflammation and demyelination associated with EAE, significantly reduced the level of glial fibrillary acidic protein (GFAP) and ionized calcium binding adapter molecule 1 (Iba-1), attenuated the activation of astrocytes and microglia, and protected blood–brain barrier (BBB) integrity by increasing the expression of key endothelial tight junction proteins. Additionally, tanshinone IIA (**1**) also inhibited the expression of some adhesion factors and chemokines involved in immune cell adhesion and migration across the BBB, thus preventing immune cell infiltration into the central nervous system [86]. Another study showed that the differentiation of T regulatory cells (Tregs) directly inhibited EAE, and tanshinone IIA (**1**) could drive Treg differentiation through a parallel mechanism by inducing dendritic cells to produce TGF-β1 and acting directly on naive CD4+ T cells, effectively inhibiting the process of EAE [87].

#### 3.3.3. Effect on Systemic Lupus Erythematosus 

Experiments have confirmed that the STAT3 signaling pathway is involved in the progression of systemic lupus erythematosus (SLE). Cryptotanshinone (**3**), as a potent inhibitor of STAT3, could reverse the elevation of STAT3 signaling in the spleen of medical research laboratory/lymphoproliferation (MRL/lpr) mice, inhibit T-cell proliferation by controlling the activation of STAT3 in vitro, and significantly attenuate autoimmune responses. In addition, the treatment with cryptotanshinone (**3**) can reduce the level of autoantibodies and inflammatory cytokines and normalize kidney structure and function, ultimately alleviating spontaneous SLE [88].

### 3.4. Improving Immune-Mediated Inflammatory Diseases

Cryptotanshinone (**3**) and tanshinone IIA (**1**) are also major components of tanshinones that improve immune-mediated inflammatory diseases (IMIDs), as displayed in Table 2.

#### 3.4.1. Effect on Psoriasis

The over-proliferation and abnormal differentiation of keratinocytes and the infiltration of inflammatory cells into the dermis and epidermis are the main features of psoriasis [89]. Danshen decoction pieces, one of the traditional Chinese medicines for psoriasis, reduced endothelin (ET) levels in the plasma of psoriasis patients and repaired damaged endothelial cells [90]. In recent years, numerous studies have been conducted on the immune and inflammatory pathway aspects of the disease, and Danshen and its active ingredients could exert anti-inflammatory, immunoregulatory, anti-keratinocyte proliferation, and pro-apoptotic effects through inhibiting the pathways of NF-κB/IκB, p38MAPK, and Th17/Treg, thereby ameliorating the psoriasis [91].

Plaque-type psoriasis is a common type of psoriasis in clinics. The effective rates of tanshinones in the treatment of psoriasis were 35.42%, 58.33%, and 68.75% at 4, 8, and 12 weeks, respectively. Moreover, the therapeutic effective rate for the tanshinones group and the Western drug retinoic acid group was basically the same, and the adverse reactions of the tanshinones group were less than those of the Western drug group, better improving patient adherence [92]. Cryptotanshinone (**3**) can treat psoriasis by inhibiting the activation of STAT3 and reducing the proliferation of keratinocytes in vivo and vitro, thereby alleviating imiquimod-induced epidermal hyperplasia [93]. The mechanism by which tanshinone IIA (**1**) ameliorated psoriasis was to strongly inhibit the proliferation of keratinocytes by inducing S-phase and G2/M-phase blockage of the cellular growth cycle and triggering apoptosis through the caspase signaling pathway [94].

#### 3.4.2. Effect on Autoimmune Hepatitis

Qin et al. showed that tanshinone IIA (**1**) played a key role in the treatment of liver injury, as evidenced by the ability of tanshinone IIA (**1**) to significantly reduce the level of plasma glutamic pyruvic transaminase (ALT) and glutamic oxaloacetic transaminase (AST), increase the ratios of CD3+, CD4+, and CD8+ T cells, and reduce liver invasion by regulating the number of T-cell subsets in immunologically mediated liver-injured mice [95]. In addition, tanshinone IIA (**1**) might protect the liver from injury by modulating pro- and anti-inflammatory cytokines. Moreover, high doses of tanshinone IIA (**1**) could also reduce inflammatory cell infiltration around the central vein of the hepatic lobule, hepatic sinusoidal congestion, hepatocyte degeneration, edema, and necrosis. Furthermore, Hao et al. conducted an analysis and concluded that tanshinone IIA (**1**) contained many effective gene targets based on network pharmacological methods that can directly target autoimmune hepatitis-related signaling pathways and inhibit the activation of NF-κB by regulating the PI3K-AKT signaling pathway, thus ameliorating hepatic inflammation in mice [96].

#### 3.4.3. Effect on Inflammatory Bowel Disease

Chen et al. demonstrated that Danshen and its tanshinone IIA (**1**) extract can treat ulcerative colitis (UC) through various pathways, such as inhibiting angiogenesis and platelet activation and aggregation; improving intestinal microcirculation, modulating intestinal bacterial flora, and restoring intestinal mucosal barrier function; regulating immune cells and inflammatory factor production; and reducing the production of NO and O_2_ free radicals [97]. Furthermore, Su et al. screened lactobacillus rhamnosus (F-B4-1) and bacillus subtillis Natto (F-A7-1) to ferment Danshen. The results showed that after gavage with the fermented Danshen, the levels of TNF-α, IL-6, and IL-1β in the serum and colon of mice were reduced, and the abnormal length and damage to the colon were restored. In short, the fermented Danshen relieved dextran sulfate sodium (DSS)-induced UC in mice more effectively than Danshen [98].

The results from Liu et al. showed that tanshinone IIA (**1**) significantly reduced the infiltration and activation of the intestinal mucosal neutrophil and the production of the colonic inflammatory cytokine for colitis in C57BL/6 mice, indicating that tanshinone IIA (**1**) could reduce inflammatory colitis in mice through a neutrophil-mediated immune response [99]. Furthermore, tanshinone IIA (**1**) ameliorated DSS-induced inflammatory bowel disease (IBD) through the pregnenolone X receptor (PXR)-mediated upregulation of exogenous metabolism and the downregulation of NF-κB signaling [100]. In addition, cryptotanshinone (**3**) could significantly reduce neutrophil infiltration in experimental colitis, improve the degree of inflammation and intestinal fibrosis in ulcerative colon, and inhibit the differentiation of Th17 cells by suppressing STAT3 signal activation, hence improving acute and chronic colitis [101]. Another study showed that cryptotanshinone (**3**) and dihydrotanshinone I (**11**) significantly increased body weight and colon length, decreased the disease activity index (DAI) score, and ameliorated pathological changes in colon tissue in rats. It also significantly inhibited the expression of inducible nitric oxide synthase (iNOS), cyclooxygenase-2 (COX-2), and NF-κBp65 and the secretion of TNF-α and IL-6 in tissues and LPS-stimulated cells and ameliorated intestinal inflammation by suppressing UC. Additionally, dehydromiltirone (**7**) improved IBD by regulating the receptor-interacting protein kinases (RIPs)–mixed lineage kinase domain-like protein (MLKL)–caspase-8 axis [102,103]. 

**Figure 3:**
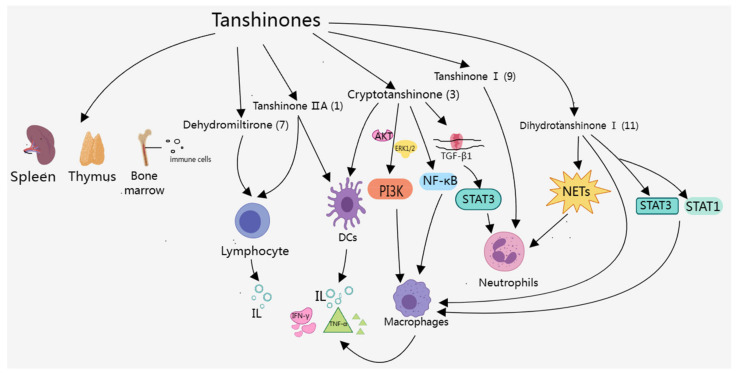
Effects of tanshinones on the immune system.

Overall, the components of tanshinones mainly control the generation and infiltration of related cells through various pathways (Figure 3), as well as the levels of immune active substances, to treat AIDs and IMIDs. Studies showed that the transcriptional regulator STAT3 had key roles in the regulation of immune responses and autoimmunity [104], as well as inhibited the expression of crucial immune activation regulators and promoted the production of immunosuppressive factors [105]. As shown in Table 2 and Figure 3, tanshinone components could alleviate RA, SLE, IBD, and Psoriasis by regulating the STAT3 signaling pathway. In additional, tanshinone IIA (**1**) was the most widely used constituent of tanshinones, which could treat RA, MS, psoriasis, AIH, and IBD. However, tanshinone IIA (**1**) had some problems, such as low water solubility and difficult absorption in solid forms, so researchers should consider designing better tanshinone IIA dosage forms.

**Table 2:** Mechanism of tanshinone components in the improvement in autoimmune and immune-mediated inflammatory diseases.

Components	Diseases	Pathways	Mechanism of Action	References
Tanshinone IIA (**1**)	RA	BMP/Wnt	Osteogenesis	[76]
		COX-2/PGE2	↓Osteoclast	[77]
		Mitochondrial	↑Caspase3/9	[78]
		Caspase	↓Procaspase3/9	
		PI3K/MAPK/AKT/mTOR/HIF	↓RA-FLSs	[80]
		Cell cycle	G2/M phase	[79]
		PI3K/AKT	↑IncRNA GAS5	[80]
		β-arrestin 2	↓TNF-α	[81]
			↓ IL-6, IL-17	
		RANKL	↓Osteoclast	[83]
	MS		↓T cells	[85]
			↓IL-17, IL-23	
		GFAP/Iba-1	↓Immune cells	[86]
		BBB	↑TGF-β1	[87]
			↑Treg	
	Psoriasis	Caspase	↓Psoriatic keratinocytes	[94]
		Cell cycle	S phase, G2/M phase	
	AIH		↓ALT, AST, ↓IFN-γ	[95]
			↓IL-2, IL-4, ↑IL-10	
		PI3K-AKT	↓ALT, AST	[96]
		NF-κB		
	IBD	ROSMPO	↓Neutrophil infiltration	[99]
			↓TNF-α	
			↓IL-1β, IL-6, IL-10	
		NF-κB		[100]
		PXR		
Cryptotanshinone (**3**)	RA	AKT	↓RA-FLSs	[72]
		MAPK		
		STAT3		
		NF-κB	↑Th17/Treg	[73]
		NF-κB	↓MMP-9	[74,75]
		ERK	↓IL-1β, IL-17α, ↓TNF-α	
	SLE	STAT3	↓T cells	[88]
	Psoriasis	STAT3	↓Psoriatic keratinocytes	[93]
	IBD	STAT3	↓Th17 cells	[101]
			↓Neutrophil infiltration	
			↓iNOS, COX-2, ↓NF-κBp65, ↓TNF-α, ↓IL-6	[102]
Dihydrotanshinone I (**11**)	UC	RIPs–MLKL–caspase-8 axis	↓iNOS, COX-2, ↓TNF-α	[103]
			↓IL-6, IL-1β	

↑ means increase; ↓ means decrease.

## 4. Effects of Salvianolic Acid Components on Immune System

**Figure 4:**
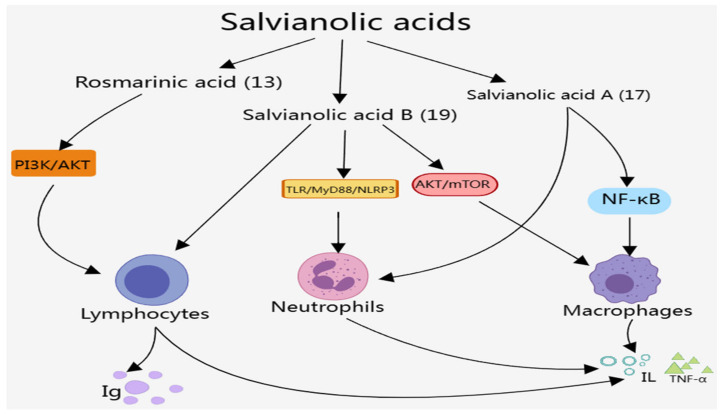
Effects of salvianolic acids on the immune system.

The effects of salvianolic acid components on the immune system are mainly manifested in regulating the number and function of macrophages, neutrophils, and lymphocytes, as well as modulating the release of immunoreactive substances such as IL, TNF, and IFN through NF-κB and other signaling pathways, as presented in Figure 4, which can play an anti-inflammatory role and maintain the stability of the immune system. Among these salvianolic acid components, most studies were on the effects of salvianolic acid A (**17**), salvianolic acid B (**19**), and rosmarinic acid (**13**) on the immune system, including regulating the number and function of immune cells, regulating the release of immunoreactive substances, and improving autoimmune diseases and immune-mediated inflammatory diseases. Here, we summarize the effects of salvianolic acid components on the immune system in detail.

### 4.1. Regulating the Number and Function of Immune Cells

#### 4.1.1. Effect on Macrophages

As displayed in Table 3, many studies showed that salvianolic acid B (**19**) could promote the conversion of macrophages from the pro-inflammatory M1 type to the anti-inflammatory M2 type by inhibiting mammalian target of rapamycin complex 1 (mTORC1)-dependent glycolysis, which contributed to attenuating the inflammatory response after myocardial ischemia/reperfusion injury and improving cardiac function [106]. Sun et al. certified that salvianolic acid B (**19**) was able to attenuate macrophage apoptosis by inhibiting the AkT/mTOR pathway and ameliorating the autophagy dysfunction of RAW264.7 macrophages. Moreover, the treatment of macrophages with salvianolic acid B (**19**) showed that the cholesterol crystal-induced release of inflammatory cytokines TNF-α and IL-6 was inhibited [107]. Salvianolic acid A (**17**) was also effective in reducing macrophage inflammatory injury and exerting anti-inflammatory effects by affecting NF-κB activity and reducing the expressions of NO, iNOS, IL-6, TNF-α, and MMP-9 [108].

#### 4.1.2. Effect on Neutrophils

It was confirmed that salvianolic acid B (**19**) could treat acute airway inflammation and oxidative stress by inhibiting TLR/TLR4/MyD88/NLRP3 pathways, significantly reducing the increase in the number and infiltration of neutrophils and macrophages in BALF induced by pm2.5, and decreasing the levels of SOD, IL-1β, and TNF-α [109]. Meanwhile, salvianolic acid A (**17**) inhibited neutrophil formation and reduced inflammation and oxidative stress, which ameliorated acute lung injury by inhibiting the scarecrow (Scr) pathway in vivo and in vitro [110].

#### 4.1.3. Effect on Lymphocytes

Salvianolic acid components had major effects on T lymphocytes and T-cell subsets, such as Th cells and cytotoxic T lymphocytes (CTLs), etc. Wang et al. [111] found that salvianolic acid B (**19**) reduced the number of NK cell-activating receptor (Nkp46) cells and cytotoxic CD8+ T cells in the placenta of female mice to a greater extent, decreased the expression of inflammatory factors and toll-like receptors in the placenta, and increased the area of the placental labyrinth, which was beneficial for the immunomodulation of the maternal–fetal interface in a mouse model of spontaneous abortion. In addition, salvianolic acid B (**19**) also significantly increased the levels of peripheral blood CD4+ T cells and CD4+/CD8+ T cells, increased the levels of serum IgA, IgG, and IgM, and reduced the levels of IL-1β, IL-6, IL-8, and TNF-α, thus improving the immune function and inflammatory response of thoracic aortic aneurysms in rats [112]. Rosmarinic acid (**13**) increased the immune organ index, improved the histopathological status of immunosuppressed mice, and enhanced the activities of lymphocytes, NK cells, and CTLs, as well as the secretion and mRNA expression of immune-related cytokines, which may play a positive role in the immune response by modulating the PI3K/AKT signaling pathway [113].

**Table 3:** Effects of salvianolic acids on the immune system.

Components	Immune Cells	Immune Active Substance	References
Salvianolic acid A (**17**)	Macrophages	↓IL-6, TNF-α	[108]
	Neutrophils		[110]
Salvianolic acid B (**19**)	Macrophages		[106]
	RAW264.7 macrophages	↓IL-6, TNF-α	[107]
	Neutrophils	↓IL-1β, TNF-α	[109]
	T cells	↓IL-6, IL-8, IL-1β, TNF-α	[111,112]
	NK cells	↑IgA, IgG, IgM	
Rosmarinic acid (**13**)	Lymphocytes		[113]
	NK cells		
	CTLs		

↑ means increase; ↓ means decrease.

### 4.2. Improving Autoimmune Diseases

#### 4.2.1. Effect on Rheumatoid Arthritis

As shown in Table 4, rosmarinic acid (**13**) and salvianolic acid B (**19**) can also improve autoimmune diseases. They improved RA mainly by inhibiting the expression of inflammatory mediators and reducing the peroxidation damage of joint tissues. Sun et al. successfully established a rat model of RA using collagen induction, after which it was found that salvianolic acid B (**19**) significantly inhibited the expression of relevant inflammatory mediators, such as TNF-α, IL-6, and prostaglandin E2 (PGE2), and improved RA [114]. 

Another study showed that salvianolic acid B (**19**) used at 20 and 40 mg/kg could inhibit the levels of inflammatory mediators in RA rats by downregulating NF-κB, and the usage of 40 mg/kg was more effective. Specifically, the expression levels of human nuclear factor κB inhibitory (pIκB-α, NF-κBp65, and IκB-α) proteins were decreased and the levels of IL-6, IL-17, IL-1β, and TNF-α were also decreased, but IL-10 was increased; the amount of neutrophil infiltration was decreased, and the levels of anti-type II collagen-specific IgG1 and IgG2a were normalized, which protected the joint tissues from harmful damage by improving endogenous antioxidant levels, thus maintaining the integrity of synovial or joint tissues and cartilage [115]. In addition, salvianolic acid B (**19**) restrained the proliferation of MH7A rheumatoid arthritis fibroblast cells and promoted their apoptosis by promoting cellular autophagy, thereby alleviating RA symptoms [116]. Rosmarinic acid (**13**) had a therapeutic effect on an experimental mouse model of RA (CIA), as evidenced by the alleviation of synovitis and reduction in COX-2-positive cells [117]. Furthermore, rosmarinic acid (**13**) induced the apoptosis of T cells, including CD3+CD25+ and CD4+CD25+ T cells, in rheumatoid arthritis patients via the mitochondrial pathway, thereby alleviating RA [118].

#### 4.2.2. Effect on Multiple Sclerosis

The abnormal expression of Th1 and Th17 cells caused EAE [119]. Dong et al. administered 30 mg/kg salvianolic acid B (**19**) into the peritoneal cavity of mice every day for 14 days and found that salvianolic acid B (**19**) significantly reduced the infiltration of inflammatory cells, limited the formation of astrocytes and macrophages, and blocked the response of Th1 cells, which effectively ameliorated EAE [120]. In addition, salvianolic acid B (**19**) might exert therapeutic effects on EAE by targeting peripheral T cells [121].

#### 4.2.3. Effect on Renal Injury in Systemic Lupus Erythematosus

More and more studies have shown that salvianolic acid A (**17**) is likely to be a promising agent for the treatment of kidney diseases. For example, Diao et al. reported that salvianolic acid A (**17**) played a significant role in improving kidney injury in rats, and the mechanism may be achieved by regulating the MAPK and TGF-β1/Smad protein (Smad) signaling pathways [122]. In addition, salvianolic acid A (**17**) could significantly reduce the levels of anti-Sm-IgG and IgM antibodies and attenuate prostaglandin-induced histopathological alterations in the kidneys of BALB/c mice by inhibiting the phosphorylation of inhibitory kappa B kinase (IKK), I-κB, and NF-κB, thereby improving renal function [123]. Another experiment showed that [124] Danshen with usage at 200, 400, 800, and 1600 mg/L could significantly inhibit the apoptosis of peripheral blood neutrophils (PMNs) as well as the production of O_2_ and NO active mediators in SLE patients cultured in vitro in a dose-dependent manner, suggesting that this may be one of the active mechanisms of Danshen in the treatment of lupus nephritis (LN).

### 4.3. Improving Immune-Mediated Inflammatory Diseases

#### 4.3.1. Effect on Psoriasis

As shown in Table 4, salvianolic acid components can improve immune-mediated inflammatory diseases. Some studies demonstrated that salvianolic acid B (**19**) could significantly reduce the area and severity of psoriasis, skin thickness, lipid peroxidation products, inflammatory markers, and keratin markers in BALB/c mice by regulating the PI3K/AKT pathway, which exhibits good anti-psoriasis properties [125]. Additionally, microemulsions of salvianolic acid B (**19**) also improved skin penetration, controlled the abnormal proliferation of keratinocytes by downregulating the IL-23/IL-17 axis, and increased skin hydration, thereby reducing psoriasis [126]. Through in vivo and in vitro studies, Jia et al. found that danshensu (**15**) inhibited abnormal epidermal proliferation in psoriasis and dose-dependently reduced the expression of Yes-associated protein (YAP), inhibited cell proliferation, induced cell cycle arrest in the G0/G1 phase, and promoted apoptosis [127]. Rosmarinic acid (**13**) attenuated imiquimod (IMQ)-induced psoriasis-like inflammation in mice by decreasing IL-23 expression, inhibiting Th17-dominated inflammation, downregulating the Janus kinase 2 (Jak2)/STAT3 signaling pathway, and inhibiting keratinocyte hyperproliferation in vivo and in vitro [128].

Chen et al. found that when 0.1% lithospermic acid (**21**) was loaded into a well-established microemulsion delivery system for in vivo administration and in vitro topical administration, lithospermic acid (**21**) was found to significantly inhibit the expression of cytokines associated with the Th-17/IL-23 axis and IMQ-induced psoriasis-like skin erythema and restore skin barrier function, thus alleviating the psoriasis [129]. 

To sum up, rosmarinic acid (**13**), salvianolic acid B (**19**), and lithospermic acid (**21**) treated the psoriasis through the IL-23/Th17 axis, as well as deceased cytokines that regulate tissue response during inflammation. However, the differences in their effects and whether they can be used together to achieve better therapeutic effects are still unknown.

#### 4.3.2. Effect on Inflammatory Bowel Disease

Wang et al. constructed an acute colitis model in rats and then injected salvianolic acid A (**17**) at 4 and 8 mg/kg/day into the tail vein of rats. The results showed that both doses of salvianolic acid A (**17**) improved the symptoms of colitis, regulated the intestinal microbiota, and reduced the expression of inflammatory factors IL-1β, monocyte chemotactic protein-1 (MCP-1), and IL-6 in a dose-dependent manner. Additionally, high doses of salvianolic acid A (17) increased colon length and improved colonic tissue structure and colonic tight junction injury [130]. In addition, salvianolic acid B (**19**) could increase the levels of SOD, catalase (CAT), and glutathione (GSH) in the colonic mucosa and decrease the levels of peroxidase myeloperoxidase (MPO) and MDA, which can improve free radical scavenging and antioxidant capacity, decrease lipid peroxidation, and attenuate colonic ulceration, thus improving UC [131]. Moreover, another study showed that rosmarinic acid (**13**) significantly ameliorated DSS-induced colonic inflammation in mice by inhibiting the expression of pro-inflammatory cytokines and inflammatory mediators through the dual inhibition of NF-κB and STAT3 activation [132]. Furthermore, rosmarinic acid (**13**)-derived nanoparticles effectively scavenged ROS, protected cells from ROS-induced damage, and passively targeted the inflamed colon to protect it from oxidative damage, thereby promoting the relief of colitis without toxicity [133]. Chitosan and nutrient sugar-encapsulated vesicles loaded with rosmarinic acid (**13**) reduced inflammation and oxidative stress by modulating NLRP3 inflammatory vesicles and reconstituting the nuclear factor erythroid 2-related factor 2 (Nrf2)/heme oxygenase-1 (HO-1) signaling pathway and decreased neutrophil infiltration and TNF-α production, thereby protecting the colonic mucosa from DSS-induced injury [134]. Additionally, total phenolic acid extract from the stems and leaves of Danshen and total tanshinone extract from the rhizomes of Danshen, alone or in combination, were effective in ameliorating DSS-induced UC in mice by inhibiting the TLR4/PIK3/AKT/mTOR pathway and suppressing the expression of TNF-α, IL-6, IL-1β, and COX-2, and the combined effect was more pronounced [135].

In summary, Rosmarinic acid (**13**), salvianolic acid A (**17**), and salvianolic acid B (**19**), as main salvianolic acid components, show much better treatment effects on the immune system autoimmune and immune-mediated inflammatory diseases than other salvianolic acids through various pathways. Salvianolic acid B (**19**) plays a key role in the regulation of a variety of immune cell subpopulations and the release of inflammatory factors, which is expected to be used in practical applications.

**Table 4:** Mechanism of salvianolic acid components in the improvement in autoimmune and immune-mediated inflammatory diseases.

Components	Diseases	Pathways	Mechanisms of Action	References
Salvianolic acid A (**17**)	LN	MAPKs		[122]
		TGF-β1/Smads		
		IKK	↓IgG ↓IgM	[123]
		I-κB		
		NF-κB		
	IBD		↓IL-1β, IL-6	[130]
			↓MCP-1	
Salvianolic acid B (**19**)	RA	NF-κB	↓IL-6, IL-17, IL-1β	[115]
			↓TNF-α	
			↑IL-10 ↓IgG1, IgG2a	
		Autophagy	↓MH7A cells	[116]
	MS		↓CD45+ cells	[120]
			↓Th1 cell responses	
			↓Microglia/Macrophages	
			↓T-lymphocytes	[121]
	Psoriasis	PI3K/AKT	↓IL-22, IL-23, IL-17A, IL-1β, IL-6	[125]
		IL-23/IL-17		[126]
	UC		↑SOD, CAT, GSH	[131]
			↓MDA, MPO	
Rosmarinic acid (**13**)	RA		↓COX-2 cells	[117]
		Mitochondrial	↓T cells	[118]
	Psoriasis	JAK2/STAT3	↓IL-23, IL-17A	[128]
		IL-23/Th 17		
	IBD	NF-κB/STAT3	↓IL-16, IL-1β, IL-22	[132]
			↓COX-2, iNOS	
		ROS	↓IL-6, IL-12, IL-1β	[133]
			↓TNF-α, IFN-γ	
		NLRP3 Nrf2/HO-1	↓Neutrophils	[134]
			↓TNF-α	
			↓IL-1β	
Danshensu (**15**)	Psoriasis	Cell cycle	G0/G1 phase	[127]
			↓YAP	
Lithospermic acid (**21**)	Psoriasis	Th-17/IL-23	↓TNF-α, IFN-γ	[129]

↑ means increase; ↓ means decrease.

## 5. Conclusions and Prospects 

As the first herbal medicine to be listed in the United States Pharmacopoeia, in recent years, the research on Danshen and its active components has mainly focused on the mechanisms of various diseases, such as neuropathic disorder, obesity, diabetic nephropathy, liver disease, and cancer. There is substantial evidence that tanshinones and salvianolic acids have immunomodulatory properties. So, tanshinones and salvianolic acids may have broad application prospects in anti-tumor immunotherapy. Moreover, tanshinones have multiple pathways for regulating immunity compared with salvianolic acid. Among these chemical constituents of Danshen, tanshinone IIA (**1**) is considered to have the best immunomodulatory properties and has been widely used in the treatment of RA, MS, Psoriasis, AIH, and IBD. These immunomodulatory properties are actually responsible for many beneficial health effects of tanshinones and salvianolic acids, making them helpful in improving autoimmune diseases and immune-mediated inflammatory diseases. However, due to the abundant components of tanshinones and salvianolic acids, the research on their immunomodulatory mechanisms is not yet systematic and complete. In particular, whether there is synergism between the components needs to be further studied. Therefore, with the development of science and technology, more chemical components will be isolated, and their immunomodulatory mechanisms need to be further studied and understood. This review provides a reference for the research and development of Danshen-related drugs and the clinical treatment of immune-related diseases. 

## Data Availability

Not applicable.
